# Apelin-13 Suppresses Neuroinflammation Against Cognitive Deficit in a Streptozotocin-Induced Rat Model of Alzheimer’s Disease Through Activation of BDNF-TrkB Signaling Pathway

**DOI:** 10.3389/fphar.2019.00395

**Published:** 2019-04-16

**Authors:** Huaiqing Luo, Yang Xiang, Xiangping Qu, Huijun Liu, Chi Liu, Guangyi Li, Li Han, Xiaoqun Qin

**Affiliations:** ^1^Department of Physiology, School of Basic Medical Science, Central South University, Changsha, China; ^2^Department of Anatomy, Histology and Embryology, Changsha Medical University, Changsha, China; ^3^Department of Physiology, School of Basic Medical Science, Changsha Medical University, Changsha, China

**Keywords:** apelin-13, Alzheimer’s disease, streptozotocin, cognition, inflammation, brain-derived neurotrophic factor

## Abstract

Alzheimer’s disease (AD), a progressive neurodegenerative disease characterized by impairments of cognitive function as a result of synaptic deficits and neuronal loss, is associated with inflammation. Apelin-13, a predominant neuropeptide with inhibiting effect on inflammation, has beneficial effects on cognition memory and neuronal damage. However, whether apelin-13 can protect neurons to ameliorate cognitive deficits in AD by inhibiting the inflammatory response remains largely unknown. To test this hypothesis, rats were intracerebroventricularly (ICV) injected with streptozotocin (3 mg/kg) alone or in combination with apelin-13 (2 μg). And tyrosine receptor kinase B (TrkB) blocker K252a (200 nM) was administrated 10 min before apelin injection. Furthermore, cognitive performance was assessed by new object recognition (NOR) and Y-maze tests. Protein expression of apelin, APJ, microglial marker (IBA1), astroglia marker (GFAP), interleukin 1 beta (IL-1β), tumor necrosis factor-α (TNF-α), synaptophysin (SYP), brain-derived neurotrophic factor (BDNF), TrkB, phospho-TrkB (p-TrkB) in the hippocampus were examined by western blotting or immunohistochemistry. And the gene expression of IBA1, GFAP, IL-1β, TNF-α, and SYP were detected by real-time quantitative polymerase chain reaction (PCR). Inflammatory disorder in the hippocampus was tested by hematoxylin and eosin (H&E) staining. The enzyme-linked immunosorbent assay (ELISA) was used to study the expression level of acetylcholine. And the activity of acetylcholinesterase was detected by Acetylcholinesterase Assay Kit. We observed that apelin/APJ signaling was downregulated in the hippocampus of rats administrated with STZ. Apelin-13 was found to significantly ameliorate STZ-induced AD-like phenotypes including congnitive deficit, cholinergic disfunction and the damage of neuron and synaptic plasticity. Moreover, apelin-13 inhibited microglia and astrocyte activation, reduced IL-1β and TNF-α expression and hippocampal BDNF/TrkB expression deficit in AD rats. Finally, apelin-13-mediated effects were blocked by TrkB receptor antagonist K252a. These results suggest that apelin-13 upregulates BDNF/TrkB pathway against cognitive deficit in a STZ-induced rat model of sporadic AD by attenuating inflammation.

## Introduction

Alzheimer’s disease (AD) is a major age-associated neurodegenerative disease characterized by progressive loss of cognitive function, inability to perform daily tasks, and finally dementia. It is estimated that the total number of people with dementia worldwide will double. The airway epithelium acts as the first line of defense against injurious insult, AD, the leading cause of dementia in the elderly, is an irreversible neurodegenerative disease characterized by progressive cognitive decline. It is reported that the prevalence of AD dementia increases exponentially with age (Ferri et al., 2005) and by 2050, about 110 million people worldwide will be living with AD (Brookmeyer et al., 2007). This clinical phenomenology has leaded to increased use of health services and heavy socioeconomic burden. Up to now, a series of drugs for the treatment of AD only play a role in relieving symptoms and can not halt its development. Therefore it still needs to explore effective therapeutic options to prevent the onset of AD and its progression.

Although the exact cause of AD remains poorly understood, it is increasingly recognized that neuroinfammation plays a crucial role in neurodegenerative disorders (Esiri, 2007). Neuroinflammation characteristiced by increased activated glial cells and proinflammatory cytokine (Kitazawa et al., 2011; Heneka et al., 2015), occurs in pathological brain regions of AD, such as hippocampus. Overactivated microglia can cause neurodegeneration by producing excessive proinflammatory cytokines and free radical (Kitazawa et al., 2011). Proinflammatory factors from activated microglia also lead to excessive activation of astrocytes to produce a multitude of inflammatory factors and cytotoxic substances and decrease the secretion of neurotrophins, such as brain-derived neurotrophic factor (BDNF) (Min et al., 2015). BDNF, a critical neurotrophin, is highly involved in neurogenesis (Lee et al., 2015), synthesis of neurotransmitters (Auld et al., 2001), synaptic plasticity and protecting neurons from damage (Cowansage et al., 2010), which can be directly related to cognitive performance. BDNF exerts above biological effects by activating tyrosine receptor kinase B (TrkB) receptor. The decreases of BDNF in the brain (Iulita et al., 2017) and serum are involved in the pathogenesis of AD (Curto et al., 2017), while intrahippocampal BDNF administration in rats shows protective role in against neuronal loss by suppressing the activation of microglia and astrocytes (Bovolenta et al., 2010). Furthermore, the neuroprotective effect of BDNF is correlated with downregulation of proinflammatory (TNF-α) and upregulation of anti-inflammatory cytokines (Jiang et al., 2011). These observations indicate that the treatments of BDNF upregulation/anti-inflammation may be novel candidates for AD therapy.

Apelin, the endogenous ligand of a formerly orphan G protein coupled receptor (GPCR), APJ, has various bioactive forms such as apelin-13, apelin-17, and apelin-36 (Cheng et al., 2012). Recently, a number of findings *in vitro* and *in vivo* have revealed that central apelin exerts regulating effects on blood pressure, feeding behavior, pituitary hormone release (Cheng et al., 2012; Xiao et al., 2018), as well as anti-depression (Dai et al., 2018), anxiolytic action (Telegdy and Jaszberenyi, 2014). Interestingly, apelin-13 a primarily active isoform specifically bound to APJ (Hosoya et al., 2000; Masri et al., 2006), is broadly disseminated in various brain tissues such as hippocampus suggesting a potential involvement of apelin/APJ signaling in cognitive capability (Han et al., 2014; Haghparast et al., 2018). Further, intracerebroventricularly (ICV) injection of apelin-13 ameliorated stress-induced memory performance deficit in rats (Dai et al., 2018). In AD patients, the serum level of apelin-13 decreased significantly (Eren et al., 2012), whereas apelin administration plays a neuroprotective role by inhibiting inflammation response, including the activation of microglia and astrocytes and the secretion of inflammatory mediators, especially TNF-α and IL-1β in animal models of brain injury (Chen et al., 2015; Xin et al., 2015). However, whether apelin-13 can ameliorate cognitive impairments of AD rats by attenuating inflammatory response as well as the underling neural mechanism are still largely unknown.

APJ combined with apelin can regulate multiple signaling pathways such as cyclic adenosine monophosphate (cAMP) that are critically correlated with BDNF transcription (O’Carroll et al., 2013; Bortolato et al., 2016), manifesting potential involvement of BDNF in anti-AD-like effect of apelin-13. Since majority of AD cases are sporadic, rather than inherited (familial), we utilize streptozotocin (STZ)-treated rats, which have been considered as the best animal model of the sporadic Alzheimer’s disease (SAD), to investigate whether ICV STZ injection decreases hippocampal apelin and APJ expression levels. Furthermore, whether apelin-13 can ameliorate STZ-induced activation of microglia and astrocytes, neuronal damage and acetylcholine level decrease to improve cognitive function through the BDNF/TrkB signaling pathway.

## Materials and Methods

### Animals

Adult male Sprague Dawley rats weighing 220–250 g were obtained from the Laboratory Animal Center of Central South University, Changsha, China. After arrival, the rats were maintained individually in a temperature- and humidity-controlled housing conditions with a 12:12 h light-dark cycle and free access to food and water. The rats were treated daily (5–6 min per rat) for 1 week to habituate them to the experimenter before behavioral testing. All experimental protocols were carried out according to the National Institutes of Health Guide for the Care and Use of Laboratory Animals approved by the Central South University at XiangYa Animal Care and Use Committee.

### Establishment of AD Model

Intracerebroventricular injection of STZ was performed as previously described (Huang et al., 2018). Rats were anesthetized with sodium pentobarbital (50 mg/kg, Sigma, St. Louis, MO, United States) and placed individually in the stereotaxic instrument (RWD Life Science Co., Ltd., Shenzhen, China). The bregma coordinates used for injection were: -1.0 mm posterior, ± 1.6 mm lateral and -3.5 mm below (Pellegrino et al., 1979). STZ from Sigma-Aldrich (St. Louis, MO, United States) was dissolved in citrate buffer (pH 4.4) before use. Three microliters of 3.0 mg/kg STZ were injected over 3 min with a Hamilton microsyringe into the bilateral ventricle on day 1 and day 3, respectively. The control rats were injected with 3 μl of citrate buffer. The needle remained in position for an additional 2 min after the injection before being withdrawn. After the last injection, one of the burr hole was filled with sterile bone wax and the other was implanted with a stainless-steel guide cannula (RWD Life Science Co., Ltd., Shenzhen, China) into the lateral ventricle and fixed to the skull with dental cement and acrylic resin which was used for the injection of apelin-13 or vehicle or K252a.

### The Expression Levels’ Assays of Apelin and APJ in Hippocampus

Two groups of rats were used to test whether STZ injection influenced hippocampal apelin and APJ expression levels. Rats (*N* = 4 per group) were killed 4 weeks after the last injection for collection of hippocampal tissues. The protein expression levels of apelin and APJ were tested by western blotting.

### Assessing the Effects of Apelin-13 on STZ-Induced Cognitive Impairment

**FIGURE 1:**
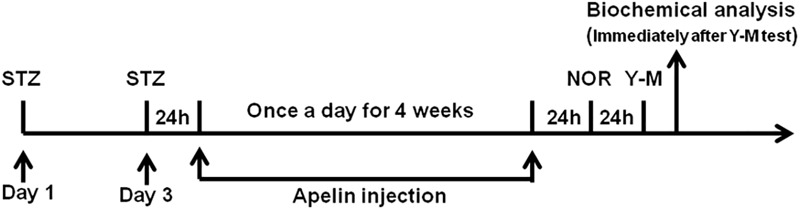
Schematic representation of the experimental designs showing the timeline of STZ administration to establish SAD model, apelin-13 administration, behavioral testing, and tissue collection for biochemical analysis. NOR, novel object recognition; Y-M, Y-maze task; Apelin, apelin-13.

Four groups were used to determine whether apelin-13 alleviates STZ-induced cognitive impairment. Rats (*N* = 10 per group) received combined injections of vehicle plus vehicle (Control group), STZ plus vehicle (STZ group), vehicle plus apelin-13 (Apelin group), and STZ plus apelin-13 (STZ +Apelin group). In detail, rats received ICV injection of STZ or vehicle (citrate buffer) bilaterally at first and third day and 24 h later were administered with apelin-13 (pGlu1) (Abbiotec LLC, San Diego, CA, United States) dissolved in sterile pyrogen-free 0.9% saline (2 μg/2 μl) or vehicle (saline, 2 μl) once a day for 4 weeks. The selection of effective dose of apelin-13 was based on pilot experiment and previous studies (Han et al., 2016; Aminyavari et al., 2019). All these rats were subjected to new object recognition (NOR) and Y-maze tests in succession 24 h after the end of apelin-13 injection. Then the brains of rats were isolated immediately after the completion of all behavioral tests for further biochemical and immunohistochemistry analysis. The timeline of experiments is showed in Figure 1.

### Assessing the Effects of TrkB Antagonist K252a on Apelin-13-Mediated Effects

Four groups were used to determine whether TrkB antagonist K252a blocks apelin-13’s effects. As described above, 24 h after STZ injection, rats (*N* = 8–10 per group) received combined injections of vehicle plus vehicle (STZ group), K252a plus vehicle (STZ+K252a group), vehicle plus apelin-13 (STZ+Apelin group) and K252a plus apelin-13 (STZ+Apelin+K252a group) once a day for 4 weeks. K252a (Sigma-Aldrich Co., St. Louis, MO, United States) dissolved in 1% DMSO (vehicle), was ICV injected (200 nM) 10 min before infusion of apelin-13 or vehicle. The dose of K252a was chosen on the basis of previous studies (Korte et al., 1995; Diogenes et al., 2004). All these rats were subjected to NOR test 24 h after the end of apelin-13 injection. Then the brains of rats were collected for further biochemical and immunohistochemistry analysis.

### Novel Object Recognition (NOR) Test

The test apparatus consisted of a black Plexiglas box (50 × 50 × 50 cm) placed in a dimly lit (45 lux) testing room. The objects used in the test were made of glass and plastic with differences in shape, color, and textures. And two objects were placed in the back corners of the box. The location and objects were counterbalanced within each group in order to avoid any preferences that the rats might have had for one of the corners or a given object. The behavioral procedure consisted of two sessions: a training session and a testing session. During the training session, the rat was allowed to explore two identical objects for 3 min and the total time spent exploring both objects was recorded. Exploration was defined as sniffing the object with the nose within 1 cm radius and/or touching it. The box as well as objects were thoroughly cleaned with 20% alcoholic solution between trials to avoid the presence of olfactory cues and then wiped with dry paper. After 24 h interval (testing session), the rat was exposed to two objects, one familiar object from the first session and a new object, for 3 min. A discrimination index (DI) used to discriminate the novel from the familiar object was calculated as follows: [time exploring the novel object (s) - time exploring the familiar (s)]/[time exploring novel (s) + familiar (s)] × 100%.

### Y-Maze Test

The Y-maze consists of three identical arms (50 cm long × 12 cm wide × 20 cm high) at an angle of 120° to each other, radiating out from a central platform (12 cm × 12 cm × 12 cm). The rat was placed at the end of one arm (facing the end of the arm) and free accessed to all three arms for a duration of 5 min. The pattern and the total number of arm entries were recorded for a 5-min testing period. An arm entry was defined as all four paws of rat completely placed on the arm. A correct alternation was defined as exploring the three arms in turn. While an incorrect alternation was scored when two arms were visited per triad of exploration. The percentage correct alternation used to determine the memory index was calculated as: the correct alternation/the total alternation × 100%.

### Sample Collection

After anesthesia with sodium pentobarbital, the brains of rats were decapitated, then the bilateral hippocampus were harvested (ice operation), and placed into a labeled tube frozen with liquid nitrogen and stored at -80°C for real-time PCR, western blotting or biochemical assays. The remaining rats were perfused and fixed for 24 h with 4% paraformaldehyde for H&E and immunochemistry.

### Total RNA Extraction and Real-Time PCR

The total RNA from hippocampal samples was isolated according to the manufacturer’s protocol. In brief, hippocampal tissues were homogenized in TRIzol reagent and then mixed with chloroform and centrifuged at 13,500 rpm and 4°C for 15 min. The upper supernatant was mixed with isopropanol in a ratio of one to one and centrifuged for 15 min at 13,500 rpm 4°C. After discarding supernatant completely, the pellets were washed with 75% ethanol for two times and then left for air drying. Finally, the mRNA pellets were dissolved with nuclease-free water. The concentration of the mRNA were detected by NanoDrop Spectrophotometer (Thermos Fisher Scientific, United States). 1 μg of mRNA was reversely transcripted to cDNA with PrimeScript^TM^ RT reagent Kit (Takara, Japan) according to manufacturer’s instruction. The qRT-PCR reactions were performed using the SYBR Green reagents (Takara, Japan) in an Applied Biosystems 7300 detection system (Bio-Rad). The data of target gene were normalized to the expression of a cellular housekeeping gene, beta actin (β-actin). The primer sequences (Sangon) used in this study are listed in Table 1.

**Table 1:** Real-time PCR primers used in this study.

Target/control gene	Primer sequences
IBA1	F 5′-ATGTCCTTGAAGCGAATGCT-3′
	R 5′-TTCTCAAGATGGCAGATCTCTT-3′
GFAP	F 5′-CCAAGATGAAACCAACCT-3′
	R 5′-CGCTGTGAGGTCTGGCTT-3′
IL-1β	F 5′-TCGGCCAAGACAGGTCGCTCA -3′
	R 5′-TGGTTGCCCATCAGAGGCAAGG -3′
TNF-α	F 5′-TCATTCCTGCTCGTGGCGGG-3′
	R 5′-CGGCTGACGGTGGGGTGAG-3′
SYP	F 5′-CTTTCTGGTACAGCCGTGAG-3′
	R 5′-ACAGGGTCCCTCAGTTCCTT-3′
β-actin	F 5′-GTCGTACCACTGGCATTGTG -3′
	R 5′-CTCTCAGCTGTGGTGGTGAA -3′

### Western Blotting

Western blotting analysis was performed as described previously (Dong et al., 2018). Briefly, tissue samples were lysed homogenizedly by RIPA lysis buffer containing 1% phenylmethanesulfonyl fluoride (PMSF) (Sigma-Aldrich), and then centrifuged at 13,000 rpm for 10 min to collect supernatants. The concentration of protein was determined using BCA Protein Assay Kit (Thermo Fisher Scientific). 5× SDS Loading Buffer was added to supernatants, and then boiled in a constant temperature metal bath for 10 min. 10% SDS-PAGE was used to separate protein. After electro transferring protein to polyvinylidene fluoride (PVDF) membranes (Millipore), the membranes were incubated with primary antibodies as follows: Anti-apelin (1497-1-AP, 1:500, Proteintech), anti-APJ antibody (20341-1-AP, 1:500, Proteintech), Anti-IBA1 (ionized calcium binding adapter molecule 1, microglia marker) (ab178847, 1:1000, Abcam), Anti-GFAP (glial fbrillary acidic protein, astroglia marker) (ab33922, 1:1000, Abcam), Anti-BDNF (SAB2108004, 1:1000, Sigma), Anti-TrkB (#4603, 1:1000, Cell Signaling Technology), Anti-p-TrkB (ab228507, 1:1000, Abcam), Anti-β-actin (A1978, 1:10000, Sigma), and subsequently reacted with relative secondary antibody conjugated with horseradish peroxidase (Anti-rabbit IgG, #7074 1:10000; Anti-mouse IgG, #7076 1:10000, Cell Signaling Technology) prior to visualizing using ECL reagents (Millipore). Film density was measured using ImageJ densitometry software and normalized against β-actin.

### Histology, H&E and Immunochemistry

Brains fixed in 4% paraformaldehyde were processed for paraffin embedding. Hematoxylin and eosin (H&E) staining was conducted on 5 μm sections according to previously published procedures (Wang et al., 2013). Immunohistochemistry (IHC) analyses were performed on hippocampus sections with the following antibodies: Anti-IL-1β (sc-12742, 1:200, Santa Cruz), Anti-TNF-α (sc-52746, 1:200, Santa Cruz), Anti-Synaptophysin (MAB5258-I, 1:200, Millipore). Zeiss Axio Scope.A1 or Zeiss Discovery.V8 Stereo microscopes (Carl Zeiss MicroImaging GmbH, Göttingen, Germany) was used and integrated with an Axio-Cam ICc3 camera (Spectra Service, Ontario, NY, United States). Images were acquired by AxioVision Rel. 4.7 software from Zeiss. The images were digitized and quantified using ImageJ.

### Biochemical Assays

The hippocampal homogenates were centrifuged and the supernatants were collected to measure the ACh levels (ab65345, abcam) and AChE activities (ab136957, abcam) using commercially available kits following the manufacturer’s guidelines.

### Statistical Analysis

All data were carried out using SPSS v22.0 and expressed as mean ± SEM. The *p*-value < 0.05 was accepted as statistically significant. Significant differences between two groups were confirmed by Student’s *t*-test, whereas differences among multiple groups were analyzed using one-way ANOVA followed by Tukey’s *post hoc* test.

## Results

### The Expression Levels of Apelin and APJ Decrease in the Hippocampus of STZ-Induced SAD Rats

**FIGURE 2:**
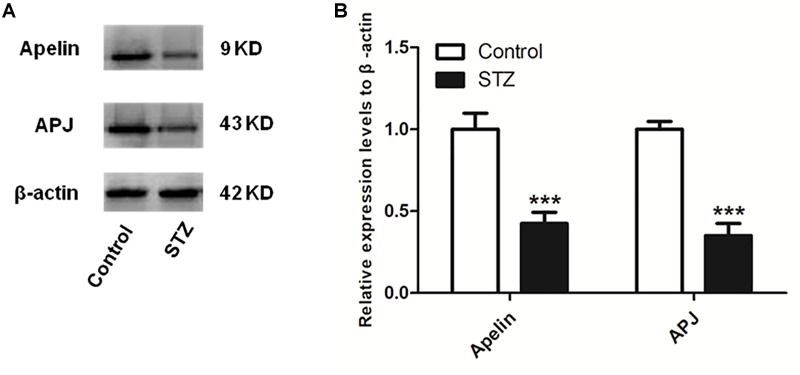
The expression levels of apelin and APJ decreases in the hippocampus of STZ-induced SAD rats. **(A)** Representative western blot analysis of apelin and APJ levels in the hippocampus. **(B)** Quantification of apelin and APJ relative to β-actin in control and STZ groups of rats. All data are presented as mean ± SEM. ^∗∗∗^*p* < 0.001 comparison to control rats, *N* = 4.

Figure 2 shows that compaired with control group, the expression levels of hippocampal apelin and APJ in the STZ group decreased significantly (apelin, *p* < 0.001, **left** panel; APJ, *p* < 0.001, **right** panel).

### Apelin-13 Improves STZ-Induced Cognitive Impairment

**FIGURE 3:**
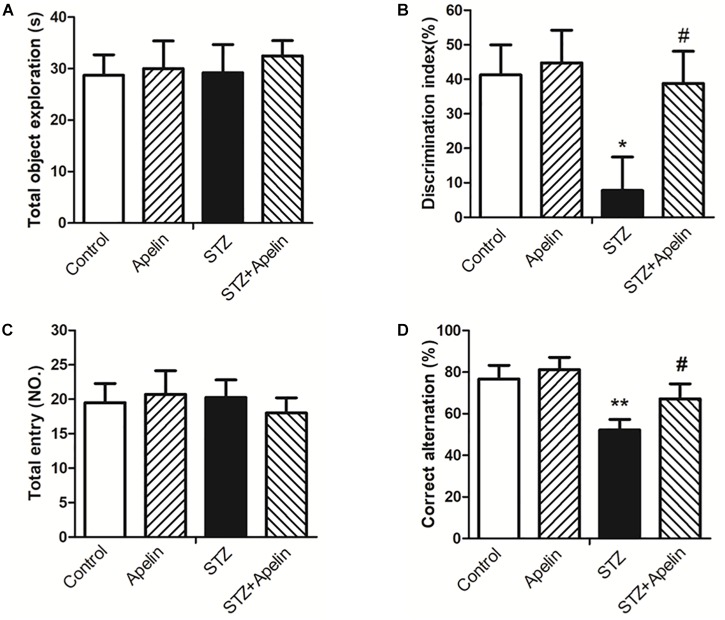
Chronic administration of apelin-13 ameliorates STZ-induced cognitive memory impairment in the NOR test **(A,B)** and Y-maze test in rats **(C,D)**. **(A)** Total exploration time spent on the training phase. **(B)** The discrimination index (DI) on the retention test phase. **(C)** Number of total entries. **(D)** Alternation ratio in rats. All data are presented as mean ± SEM. ^∗^*p* < 0.05, ^∗∗^*p* < 0.01 versus control group; ^#^*p* < 0.05 versus STZ group, *N* = 10 in each group.

In the NOR test, statistical analysis showed no significant difference in exploring the identical objects between the four groups during the training session (Figure 3A; *F*(3,36) = 0.127, *p* > 0.05), suggesting that chronic treatment of apelin-13 does not affect the exploration of the objects. However, the DI was significantly different between the four groups [Figure 3B; *F*(3,36) = 6.093, *p* < 0.01]. *Post hoc* analysis showed that the STZ group presented lower DI relative to the control group (*p* < 0.05). There was no significant difference in DI between the control group and the apelin group (*p* > 0.05). The STZ-injected rats treated with apelin-13 (STZ+ Apelin group) showed higher levels of DI than those in the STZ group (*p* < 0.05). These data indicate that chronic administration of apelin-13 improves STZ-induced memory impairment in the NOR task in rats.

In the Y-maze test, no significant difference in the number of total entries was observed between the four groups [Figure 3C; *F*(3,36) = 0.258, *p* > 0.05], indicating that chronic administration of apelin-13 does not affect locomotor activity. Analysis of the alternation ratio during the test session showed a significant difference between the four groups [Figure 3D; *F*(3,36) = 8.261, *p* < 0.001]. *Post hoc* comparisons showed that compared with the control group, the STZ group showed less levels of the alternation ratio (*p* < 0.01). There was no significant difference in the alternation ratio between the control group and the apelin group (*p* > 0.05). The STZ+ Apelin group showed higher levels of the alternation ratio than STZ group (*p* < 0.05). These results indicate that chronic administration of apelin-13 improves STZ-induced memory impairment in the Y-maze task in rats.

### Apelin-13 Inhibits STZ-Induced Glial Cell Activation and Inflammatory Expression

**FIGURE 4:**
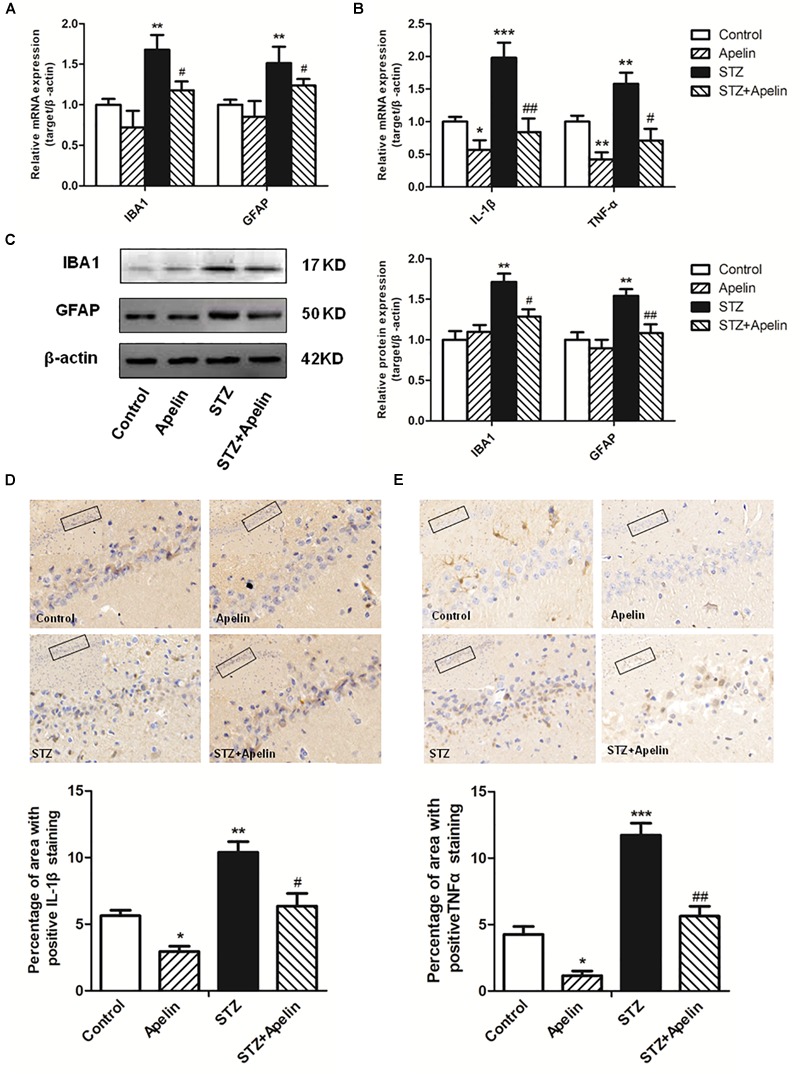
Chronic administration of apelin-13 ameliorates STZ-induced glial cell activation and inflammatory factors expression in the hippocampus. **(A)** mRNA expressions of microglial marker IBA1 and astrocyte marker GFAP in different groups of rats hippocampus. **(B)** mRNA expressions of inflammatory factors IL-1β and TNF-α in the rat hippocampus. **(C)** Representative western blot analysis of IBA1 and GFAP levels in the hippocampus and quantification relative to β-actin in different groups of rats. **(D)** Immunoreactivity of IL-1β in the hippocampus of rats and quantitative results in different groups. **(E)** Immunoreactivity of TNF-α in the hippocampus of rats and quantitative results in different groups. Images of each group were obtained at 10× and 20× magnification. All data are presented as mean ± SEM. ^∗^*p* < 0.01, ^∗∗^*p* < 0.01, ^∗∗∗^*p* < 0.001 versus control group; ^#^*p* < 0.05, ^##^*p* < 0.01 versus STZ group, *N* = 4 in each group.

Neuroinflammation attributed to the activation of glial cells and the release of numerous cytokines, plays a fundamental role in the neuropathological changes that are observed in AD. To explore the possible mechanism underling apelin-13-mediated anti-SAD-like effects, we examined whether apelin-13 ameliorated STZ-induced the activation of microglia (IBA1) and astrocyte (GFAP) and the expression of inflammatory factors (IL-1β and TNF-α). Hippocampal IBA1 and GFAP mRNA expression levels were significantly increased in the STZ group when compared to those in the control group (IBA1, *F*_3,12_ = 22.508, *p* < 0.001, *post hoc* tests, *p* < 0.01; GFAP, *F*_3,12_ = 18.164, *p* < 0.001, *post hoc* tests, *p* < 0.01) (Figure 4A), while apelin-13 partially but significantly attenuated the up-regulation of IBA1 and GFAP mRNA in the STZ group (*P* < 0.05, respectively). Consistent with the gene expression results, western blot analyses also showed a significantly increased IBA1 and GFAP protein expression levels in the STZ group (IBA1, *F*_3,12_ = 9.265, *p* < 0.01, *post hoc* tests, *p* < 0.01; GFAP, *F*_3,12_ = 7.578, *p* < 0.01, *post hoc* tests, *p* < 0.01) (Figure 4C), and this increase was ameliorated by apelin-13 (IBA1, *p* < 0.05; GFAP, *p* < 0.01). At the gene expression levels, STZ-injected rats showed significantly enhanced IL-1β and TNF-α expression levels in the hippocampus (IL-1β, *F*_3,12_ = 48.407, *p* < 0.001, *post hoc* tests, *p* < 0.001; TNF-α, *F*_3,12_ = 27.069, *p* < 0.001, *post hoc* tests, *p* < 0.01) (Figure 4B). These increases were attenuated by apelin-13 (IL-1β, *p* < 0.01; TNF-α, *p* < 0.05). Additionally, we observed that apelin-13 declined hippocampal IL-1β and TNF-α mRNA expression in non-STZ injected rats (IL-1β, *p* < 0.05; TNF-α, *p* < 0.01). Paralleling the gene expression data, immunohistochemical (IHC) staining of rats hippocampus also showed the expression levels of IL-1β(Figure 4D; *F*_3,12_ = 24.526, *p* < 0.01, *post hoc* tests, *p* < 0.01) and TNFα (Figure 4E; *F*_3,12_ = 48.743, *p* < 0.001, *post hoc* tests, *p* < 0.001) in the STZ group were higher than those in the control group, which were attenuated by apelin-13 (IL-1β, *p* < 0.05; TNF-α, *p* < 0.01). In addition, apelin-13 itself decreased hippocampal protein expression of IL-1β (*p* < 0.05) and TNF-α (*p* < 0.05).

### Apelin-13 Attenuates STZ-Induced Neuron Damage

**FIGURE 5:**
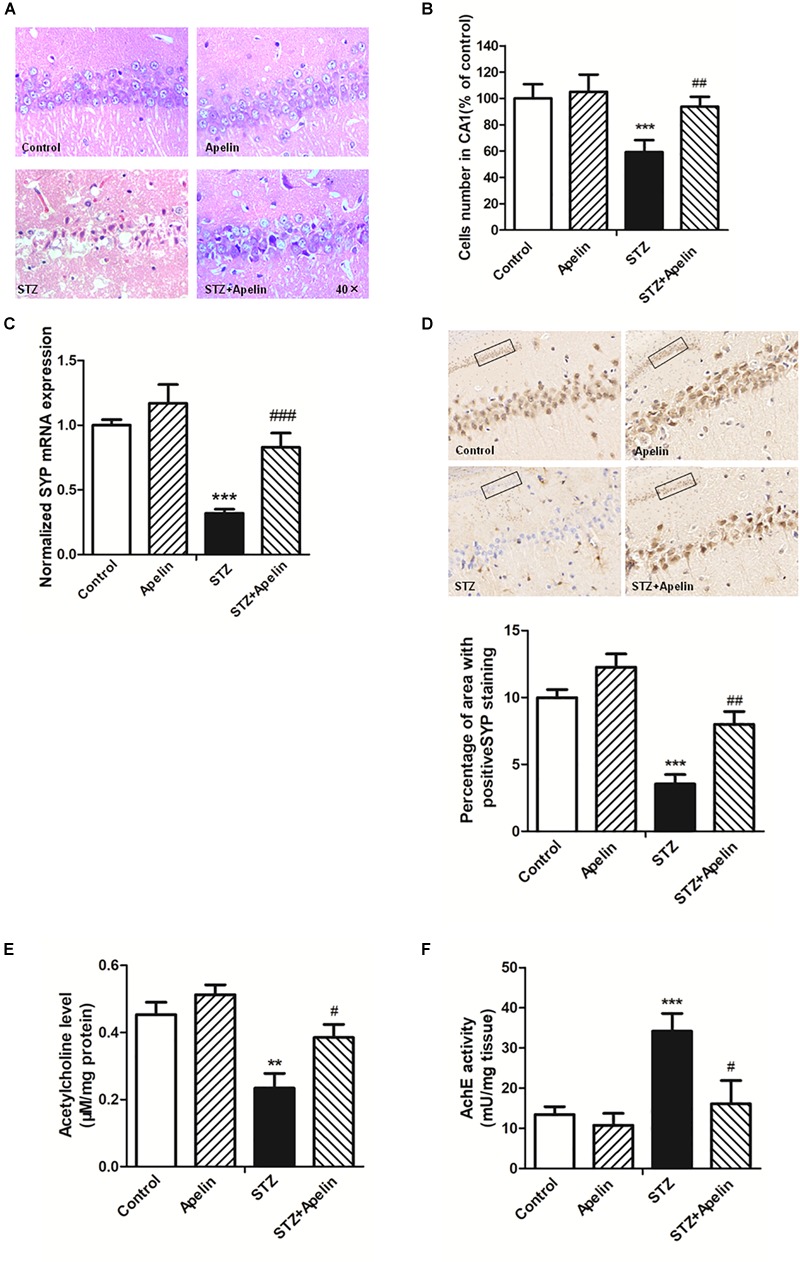
Chronic administration of apelin-13 ameliorates STZ-induced neuron damage, synaptic disorder, and cholinergic dysfunction in the hippocampus. **(A)** H&E staining of hippocampal tissues from different groups of rats. All images were obtained at 40× magnification. **(B)** Statistical analyses for the number of cells in the CA1 hippocampal region. *N* = 4 in each group. **(C)** mRNA expressions of SYP in different groups of rats hippocampus. *N* = 4 in each group. **(D)** Immunoreactivity of SYP in the hippocampus of rats and quantitative results in different groups. Images of each group were obtained at 10× and 20× magnification. *N* = 4 in each group. **(E)** The levels of hippocampal ACh in different groups. *N* = 6 in each group. **(F)** The activity of hippocampal AChE in different groups. *N* = 6 in each group. All data are presented as mean ± SEM. ^∗∗^*p* < 0.01, ^∗∗∗^*p* < 0.001 versus control group; ^#^*p* < 0.05, ^##^*p* < 0.01, ^###^*p* < 0.001 versus STZ group.

High levels of inflammation can act on neurons to result in pathologic changes, which lead to cognitive impairment (Shobin et al., 2017). To determine whether apelin-13 ameliorated STZ-induced neuron damage, hippocampal neuron structure and synaptic plasticity were examined. H&E staining results showed that hippocampal cells in the control group exhibited a regular arrangement, distinct edges and a clear nucleus and nucleolus (Figure 5A). While in the STZ group, hippocampal cells showed arrangement irregular, structure ambiguous, nucleus shrinkage and deep staining, which could be significantly attenuated by apelin-13. There was no significant difference in the neuron structure between the apelin and control groups. In addition, a one-way ANOVA (Figure 5B) revealed that the number of cells in the STZ group showed a significantly smaller than that in the control group (*F*_3,12_ = 25.471, *p* < 0.001, *post hoc* tests, *p* < 0.001). This decrease was prominently attenuated by apelin-13 (*p* < 0.01). There was no significant difference in the number of cells between the apelin and the control groups (*p* > 0.05). These results indicate that apelin-13 attenuates STZ-induced hippocampal cellular loss in rats.

Real-time PCR and immunohistochemistry were used to detect the expression of synaptophysin (SYP), a protein which can be used to reflect synaptic transmission and synaptic plasticity. The results showed that the gene level of SYP in the STZ group were significantly lower than that in the control group (Figure 5C, *F*_3,12_ = 34.223, *p* < 0.001, *post hoc* tests, *p* < 0.001), which suggests that SYP decrease may be involved in the degradation of nerve function in SAD. Compared with the STZ group, SYP expression in the STZ+Apelin groups was significantly increased (*p* < 0.001). While no significant difference was found in the expression of SYP between the apelin and control groups (*p* > 0.05). Immunohistochemical (IHC) staining analysis (Figure 5D) confirmed the qPCR findings showing that the protein expression level of SYP was decreased in the STZ versus control group (*F*_3,12_ = 42.302, *p* < 0.001, *post hoc* tests, *p* < 0.001), but was reversed in the STZ+Apelin versus STZ group (*p* < 0.01). No significant difference between apelin and control groups. These results suggest that apelin-13 can upregulate the expression of SYP of STZ rats. Therefore, one can explain the effect of apelin-13 on the cognitive impairment of STZ rats.

To figure out whether apelin-13 has effect on STZ- induced changes in the level of ACh (Figure 5E) and activity of AChE (Figure 5F) in the hippocampus, we performed the ACh ELISA assay and acetylcholinesterase assay. Statistical analysis revealed that there were significant changes in the ACh levels [*F*(3,20) = 15.448, *p* < 0.01] and AChE activity [*F*(3,20) = 37.089, *p* < 0.001] between groups. *Post hoc* tests showed that STZ significantly decreased ACh levels (*p* < 0.01) and increased AChE activity (*p* < 0.001), which were reversed by apelin-13 (*p* < 0.05, respectively). However, apelin-13 single treatment did not alter the ACh levels and AChE activity (*p* > 0.05, respectively).

### BDNF/TrkB Signaling Is Required for Apelin-13-Mediated Effects

**FIGURE 6:**
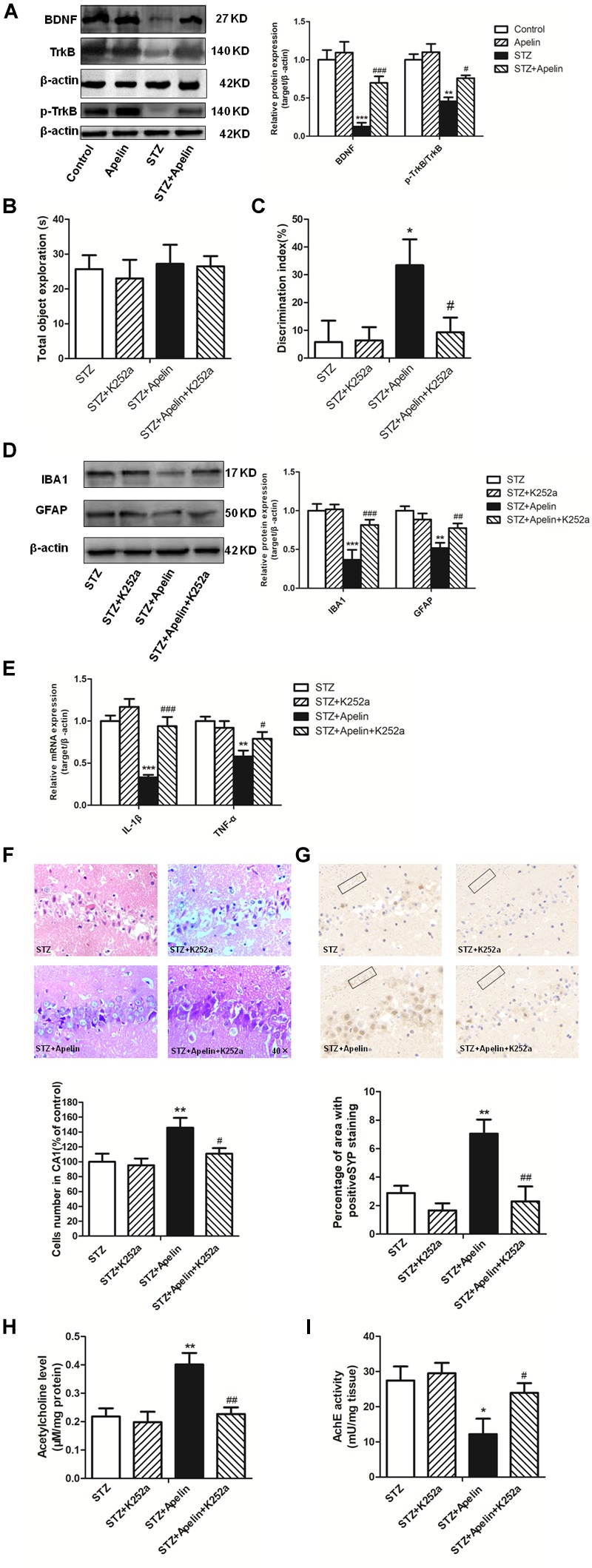
BDNF/TrkB signaling is required for apelin-13-mediated effects. **(A)** Representative western blot analysis of BDNF and p-TrkB levels in the hippocampus and quantification relative to β-actin in different groups of rats. ^∗∗^*p* < 0.01, ^∗∗∗^*p* < 0.001 versus control group; ^#^*p* < 0.05, ^###^*p* < 0.001 versus STZ group. *N* = 4 in each group. **(B)** Total exploration time spent on the training phase in NOR test. STZ group: *N* = 8; STZ+K252a group: *N* = 8; STZ+Apelin group: *N* = 10; STZ+Apelin+K252a group: *N* = 9. **(C)** The DI on the retention test phase in NOR test. **(D)** Representative western blot analysis of IBA1 and GFAP levels in the hippocampus and quantification relative to β-actin in different groups of rats. *N* = 4 in each group. **(E)** mRNA expressions of inflammatory factors IL-1β and TNF-α in the rats hippocampus. *N* = 4 in each group. **(F)** H&E staining of hippocampal tissues from different groups of rats and statistical analyses for the number of cells in the CA1 hippocampal region. All images were obtained at 40× magnification. *N* = 4 in each group. **(G)** Immunoreactivity of SYP in the hippocampus of rats and quantitative results in different groups. *N* = 4 in each group. Images of each group were obtained at 10× and 20× magnification. **(H)** The levels of hippocampal ACh in different groups. *N* = 6 in each group. **(I)** The activity of hippocampal AChE in different groups. *N* = 6 in each group. All data are presented as mean ± SEM. ^∗^*p* < 0.05, ^∗∗^*p* < 0. 01, ^∗∗∗^*p* < 0.001 versus STZ group; ^#^*p* < 0.05, ^##^*p* < 0.01, ^###^*p* < 0.001 versus STZ+Apelin group.

To investigate whether BDNF signaling is required for apelin-13-mediated effects, we first detected the expression levels of BDNF and TrkB phosphorylation in the hippocampus. Hippocampal BDNF levels in the STZ group exhibited a significant decrease (Figure 6A, *F*_3,12_ = 42.276, *p* < 0.001, *post hoc* tests, *p* < 0.001), which was partly ameliorated by apelin-13 (*p* < 0.001). In addition, apelin-13 itself did not make effect on the expression level of hippocampal BDNF (*p* > 0.05). Paralleling BDNF, TrkB phosphorylation in the STZ group was significantly decrease as compared with that in the control group (Figure 6A; *F*_3,12_ = 15.337, *p* < 0.01, *post hoc* tests, *p* < 0.01), while apelin-13 markedly ameliorated this down-regulation (*p* < 0.05).

Further experiments were designed to examine the influence of TrkB receptor antagonist K252a on apelin-13-mediated effects. The results showed that K252a pretreatment blocked apelin-13-mediated effects of STZ-induced decreased DI (Figure 6B,C; *F*_3,31_ = 8.407, *p* < 0.01, *post hoc* tests, *p* < 0.05), increased the activation of microglia (*F*_3,12_ = 28.216, *p* < 0.001, *post hoc* tests, *p* < 0.001) and astrocyte (*F*_3,12_ = 21.247, *p* < 0.001, *post hoc* tests, *p* < 0.01) (Figure 6D) and the expression of IL-1β (*F*_3,12_ = 24.353, *p* < 0.001, *post hoc* tests, *p* < 0.001)and TNFα (*F*_3,12_ = 11.426, *p* < 0.01, *post hoc* tests, *p* < 0.05) (Figure 6E), damaged neuron (Figure 6F; *F*_3,12_ = 14.473, *p* < 0.01, *post hoc* tests, *p* < 0.05), decreased SYP expression (Figure 6G; *F*_3,12_ = 49.589, *p* < 0.001, *post hoc* tests, *p* < 0.01), decreased ACh levels (Figure 6H; *F*_3,20_ = 24.673, *p* < 0.01, *post hoc* tests, *p* < 0.01) and increased AchE activity (Figure 6I; *F*_3,20_ = 8.369, *p* < 0.01, *post hoc* tests, *p* < 0.05). K252a pretreatment alone did not affect DI, microglia and astrocyte activition, IL-1β, TNFα and SYP expression, neuron damage, ACh levels and AchE activity (all, *p* > 0.05).

## Discussion

The current study verified that hippocampal apelin and APJ signaling downregulated dramatically in the STZ-induced SAD rats. Apelin-13 ameliorated congnitive impairment of STZ-treated rats in the NOR and Y-maze tests. Furthermore, apelin-13 attenuated STZ-induced hippocampal glial activation and the release of IL-1β and TNF-α, hippocampal BDNF and TrkB expression deficit, neuronal damage and cholinergic dysregulation. Finally, apelin-13-mediated effects were blocked by TrkB antagonist K252a.

The relationship between apelin/APJ signaling and AD still receives little attention in clinic. A study showed that serum apelin decreased significantly in AD patients (Eren et al., 2012). In line with this finding, an downregulation of hippocampal apelin and APJ levels were found in STZ-induced SAD rats. Furthermore, apelin-13 administration ameliorated STZ-induced AD-like phenotypes, suggesting that enhancement of hippocampal apelin/APJ signaling may indicate a new treatment approach to AD.

Intracerebroventricularly injection of STZ, a glucosamine-nitrosourea chemical compound, can lead to impairments of cerebral glucose and energy metabolism, increase of acetylcholinesterase (AChE) activity, hyperphosphorylation of tau proteins, Aβ accumulation (Biasibetti et al., 2013; Halawany et al., 2017), reduction of hippocampal synaptic transmission, oxidative stress and inflammation (Shoham et al., 2007; Salkovic-Petrisic et al., 2011), which finally lead to cognitive deficits (Abbasi et al., 2018). These effects parallel sporadic dementia of AD in humans (Hoyer et al., 1991). Thus, ICV STZ injection in rodents could serve as a reliable model for investigating the mechanism and therapeutic intervention of SAD (Salkovic-Petrisic and Hoyer, 2007). As expected, it was found that rats subjected to STZ exhibited a cognitive deficit in the NOR and Y maze tasks which were usually used to assess the cognitive memory in AD research (da Costa et al., 2017; Adeli et al., 2018; Bhuvanendran et al., 2018). After treatment with apelin-13, cognitive memory impairment induced by STZ was attenuated that was indicated by appearing increase DI in the NOR test and correct alternation in the Y maze test. Similarly, in animal, several studies have suggested that apelin/APJ signaling is involved in the regulation of cognition memory. For example, it has been reported that apelin-13 improves stress-induced cognition memory impairment in rats (Li et al., 2016), and significantly reduces cognitive impairments in Parkinsonism rats (Haghparast et al., 2018). Moreover, a recent study found that apelin-13 ameliorated memory impairments of AD induced by Aβ25-35 through inhibition of autophagy and suppression of apoptosis (Aminyavari et al., 2019). Hence, these outcomes further support that apelin-13 has an anti-amnesic effect in STZ-induced SAD model.

A growing body of data have revealed that the activity of microglia and astrocytes in AD increases. Overactivated microglia and astrocytes play a vital role in neuroinflammation via secretion of cytokines and chemokines (Yucesoy et al., 2006) such as IL-6 and IL-1β (Blum-Degen et al., 1995), TNF-α (Fillit et al., 1991), intercellular adhesion molecule I (ICAM-I), which can directly or indirectly damage neurons and cause neurodegeneration (Vivekanantham et al., 2015; Walker, 2018). While in animal transgenic AD models, anti-inflammatory treatments can reduce AD pathology (McGeer and McGeer, 2007). These observational studies serve as the bedrock to support that neuroinflammation plays a crucial role in developing AD. And inhibiting neuroinflammation may be the key to AD treatment (McGeer et al., 2016; Mishra and Brinton, 2018). Moreover, the relationship between inflammation and STZ-induced cognitive impairment has been widely acknowledged (Chu et al., 2014). Cognitive impairments caused by STZ administration will be attenuated if the excessive activation of the inflammatory response has been corrected (Dik et al., 2005). Consistent with these observations, the present results indicated that STZ exposure upregulated microglia and astrocytes activity as well as IL-1β and TNF-α levels, and these upregulation were ameliorated by apelin-13. These findings indicate that the beneficial effects of apelin-13 on STZ-induced AD-like phenotypes are closely related to its ameliorating actions on neuroinfammation by modulating glial activity.

Moreover, hippocampus as a primary brain area in AD, showed prominent neuronal and synaptic loss (Sultana et al., 2010), which can be directly related to cognitive deficits (Lee et al., 2015). It has been reported that excessive neuroinflammation caused by astrocytes and microglia activation plays a pivotal role in the cascade of events by stress-activated signal transduction pathways (Stamouli and Politis, 2016; Xu et al., 2017). IL-1β can increase the excitotoxic damage of neurons, meanwhile the antagonist of IL-1 receptor decreases neuronal death (Hailer et al., 2005). In APP/PS1 transgenic AD mice, anti-TNF-α reduced the number of amyloid plaques and tau phosphorylation and related pathologies (Shi et al., 2011). In agreement with previous findings, STZ-induced SAD rats indeed presented histopathological changes including hippocampal neuron injury and loss and synaptic plasticity loss marked by the decrease of SYP expression. Acetylcholine, a neurotransmitter involved in the regulation of cognitive function, presents a reduced concentration in the hippocampus in AD (Guo et al., 2016). One cause of ACh decrease may be the loss of cholinergic neurons induced by STZ. The other one is that STZ increases AChE activity which expedites ACh breakdown. All above pathological changes were ameliorated by apelin-13. Likewise, previous study manifests that apelin-13 exerts a protective role for neurons against cerebral ischemia/reperfusion (I/R) injury through inhibiting the expression of inflammatory factors (IL-1β, TNF-α, and ICAM-I) and the activation of microglia and astrocytes (Xin et al., 2015). Therefore, we proposed that the mechanism responsible for the anti-amnesic effect of apelin-13 in STZ-induced SAD model may be correlative with the amelioration of neuron damage caused by excessive inflammation.

Population studies have demonstrated the role of BDNF in the cognitive dysfunction of AD and its treatment (Song et al., 2015; Iulita et al., 2017). The STZ exposure inhibited hippocampal BDNF expression and signaling, which were consistent with previous studies that BDNF levels declined in animals or patients with neurodegenerative diseases in this region (Lee et al., 2005; Sarkaki et al., 2018). Hippocampal infusion of apelin-13 induces a similar behavioral profile to that following intrahippocampal BDNF infusion (Xu et al., 2018; Aminyavari et al., 2019). These findings provide evidence that apelin-13 possibly exerts a neurotrophic action in the hippocampus to mediate its anti-AD-like effects. This inference was confirmed by further observations. Apelin-13 administration ameliorated STZ-induced decrease of hippocampal BDNF and p-TrkB expression, and TrkB blocker K252a antagonized apelin-13-mediated amelioration of STZ-induced cognitive deficits, which is consistent with previous data showing the requirement for BDNF in anti-AD-like responses (Tapia-Arancibia et al., 2008). Apelin-13 may promote BDNF production by activating PI3K/Akt, ERK, or eNOS signaling pathways (O’Carroll et al., 2013; Banoujaafar et al., 2016; Yang et al., 2018), which needs to be verified in future study.

Inflammation can give rise to neuron damage which is directly related to cognitive impairments (Holmes, 2013). While BDNF binding to high-affinity TrkB receptors, suppresses neuroinfammation and protects neurons through several intracellular pathways, including the mitogen-activated protein kinase (MAPK) and the phosphoinositide 3-kinase (PI3 K)/Akt (Huang and Reichardt, 2003; Xu et al., 2018). Also, TrkB reduction can induce neuroinflammation response and neuron apoptosis (Mamidipudi and Wooten, 2002; Dong et al., 2018). K252a pretreatment blocked apelin-13’s ameliorating effects on STZ-induced activation of microglia and astrocytes and increase of inflammatory factor IL-1β and TNF-α, neuron and synapse damage, AChE activity increase and ACh levels decrease in the hippocampus, indicating that BDNF/TrkB signaling is involved in apelin-13-mediated effects on neuroinflammatory activation and neuron damage. Collectively, it is possible that apelin-13 upregulates BDNF to reduce neuron damage by modulating glial cell activation. Additionally, we found that apelin-13 did not influence BDNF and TrkB expression levels in non-STZ infused rats, suggesting the neuroprotective role of apelin-13 by regulation of BDNF/TrkB pathway mainly occurs in pathological conditions.

## Conclusion

The data in this manuscript demonstrates that apelin-13 upregulates BDNF against STZ-induced congnitive impairment by suppressing glial cell activity and inflammatory factors release. This suggests apelin signaling may be a new target in the treatment of AD.

## Ethics Statement

All experimental protocols were carried out according to the National Institutes of Health Guide for the Care and Use of Laboratory Animals approved by the Central South University at XiangYa Animal Care and Use Committee.

## Author Contributions

XqQ and LH conceived the study and contributed to its experimental design. HqL carried out the laboratory experiments, analyzed the data, and drafted the manuscript. YX, XpQ, HjL, CL, and GyL analyzed and discussed the data. XqQ and LH edited the manuscript. All authors read and approved the final manuscript.

## Conflict of Interest Statement

The authors declare that the research was conducted in the absence of any commercial or financial relationships that could be construed as a potential conflict of interest.
